# Multi-scale feature refinement network for lower limb fracture detection in X-ray images

**DOI:** 10.3389/fmed.2026.1880371

**Published:** 2026-07-02

**Authors:** Zhengguo Wan, Yanling Wang, Rong Tang, Ke Zhang, Penghua Liu, Zheyu Zhao, Yujie Shi, Huaran Huo

**Affiliations:** 1 CT and MRI Department, Handan First Hospital, Handan, China; 2 College of Information Engineering, Sichuan Agricultural University, Ya’an, China; 3 The First Clinical Medical College, Guangdong Medical University, Zhanjiang, China; 4 Orthopedic Department, Handan First Hospital, Handan, China

**Keywords:** attention mechanism, lower limb fracture, multi-scale, object detection, precision medicine

## Abstract

**Introduction:**

Lower limb fractures are common orthopedic emergencies, and rapid and accurate diagnosis of such fractures is essential for clinical practice. However, challenges such as large variations in lesion scales and ambiguous boundaries between fracture foreground and background hinder precise detection of lower limb fractures.

**Method:**

To address these challenges, we propose a multi-scale feature refinement network, MFRNet. Two innovative modules are introduced: the Adaptive Feature Perception Block (AFPB) enhances fracture-related features and suppresses background noise via a bottleneck structure and a lightweight channel-spatial attention mechanism. The Multi-Scale Dilated Attention Module (MSDAM) expands the receptive field through parallel dilated convolutions and multi-head attention mechanism, thereby capturing rich multi-scale contextual information.

**Results:**

Extensive experimental results on the lower limb fracture X-ray dataset demonstrate that MFRNet achieves 89.1% mAP and 52.2% mAP50-95 with only 3.8 M parameters, outperforming current mainstream object detection models.

**Discussion:**

The proposed MFRNet strikes a favorable balance between high detection accuracy and parameter efficiency, offering clinical practical value for computer-aided diagnosis of lower limb fractures. Future work will focus on multi-center validation, fracture type classification, and mobile deployment.

## 1 Introduction

Lower limb fractures are among the most common types of injuries in orthopedic emergency settings, with a particularly high incidence in traffic accidents, sports injuries, and falls among the elderly (1). According to statistics, lower limb fractures account for more than 30% of all body fractures, among which fractures of the femur, tibia and fibula, and the ankle joint are the most prevalent (2). Delayed or inaccurate diagnosis and treatment planning can lead to fracture malunion, delayed union, or even nonunion, severely impairing patients’ walking function and quality of life (3). Therefore, rapid and precise fracture detection is of critical importance for clinical decision-making and prognosis improvement.

Currently, plain X-ray radiography remains the first-line imaging modality for lower limb fractures due to its low cost, rapid operation, and low radiation dose (4). However, lower limb X-ray images are characterized by complex anatomy, low contrast between bone and soft tissue, and diverse fracture line morphologies. Even experienced orthopedic surgeons may find it difficult to avoid missed or incorrect diagnoses. In particular, occult fractures or subtle cracks are easily overlooked during routine reading. Hence, developing an efficient and robust computer-aided detection (CAD) system to help clinicians rapidly localize fractures holds significant clinical value.

In recent years, deep learning-based object detection algorithms have made substantial progress in medical image analysis (5). Among them, the YOLO (You Only Look Once) (6) series, known for its excellent trade-off between speed and accuracy, has been widely applied to tasks such as pulmonary nodule detection, breast tumor identification, and fracture detection. In the context of fracture detection, Wang et al. explored the performance of YOLO-series algorithms, highlighting the advantages of deep learning architectures (7). Zhang et al. (8) proposed a novel wrist fracture detection method, PEYOLO, based on multi-scale feature enhancement. Du et al. (9) introduced ASC-YOLO, a novel fracture detection method that effectively addresses the issue of missed small-target fractures. Nevertheless, for fracture detection in lower limb X-ray images, existing YOLO models still face the following challenges: (1) fracture region scales vary dramatically, ranging from fine cracks to large segmental fractures spanning long bones, which leads to insufficient multi-scale feature fusion; (2) the grayscale values near the boundaries between bone and surrounding soft tissue are similar, and background noise is substantial, which degrades feature discriminability. These issues limit the generalizability of existing models in real-world clinical scenarios.

To overcome the above limitations, we propose a multi-scale feature refinement network, termed MFRNet, for lower limb fracture detection in X-ray images. The specific contributions are as follows:

(1) We design a novel Adaptive Feature Perception Block (AFPB). By integrating a bottleneck structure with a lightweight channel-spatial attention mechanism and residual connections, AFPB effectively enhances fracture-related features while suppressing background noise, thereby improving feature discriminability.
(2) We introduce a Multi-Scale Dilated Attention Module (MSDAM), which leverages parallel dilated convolutions with different dilation rates and multi-head attention mechanism to effectively enlarge the receptive field, thus enhancing the model’s multi-scale context extraction capability.
(3) We propose a novel lower limb fracture detection algorithm, MFRNet, which adaptively mines multi-scale lower limb fracture features by enhancing feature responses in fracture regions and capturing multi-scale contextual information. Comprehensive experimental results demonstrate that MFRNet significantly outperforms current state-of-the-art algorithms.

## 2 Related work

### 2.1 Yolo

Object detection is one of the core tasks in computer vision, aiming to locate objects of interest in an image and recognize their categories (10). Since the rise of deep learning, convolutional neural network (CNN)-based object detection algorithms have been broadly divided into two-stage detectors (e.g., Faster R-CNN (11)) and single-stage detectors (e.g., the YOLO series) (12). Two-stage methods first generate candidate regions and then perform classification and regression, achieving high accuracy but slower speed. Single-stage methods directly regress bounding boxes and categories, offering significant advantages in real-time performance.

YOLO was first proposed by Redmon et al. (13), with its core idea being to unify the detection task as a regression problem: dividing the image into a grid, where each grid predicts bounding boxes and class probabilities. YOLOv2 introduced anchor box mechanism and multi-scale training, improving detection accuracy and generalization. YOLOv3 (14) adopted the Feature Pyramid Network (FPN), significantly enhancing small object detection performance. YOLOv4 (15) integrated various optimization strategies such as CSPNet, PANet, and Mosaic data augmentation, achieving an excellent balance between speed and accuracy. YOLOv5 (16) further optimized the network architecture, data augmentation, and hyperparameters, becoming one of the most widely used versions in industrial applications.

In recent years, the YOLO series has continued to evolve. YOLOv6 (17) introduced the EfficientRep backbone and Rep-PAN neck, balancing deployment-friendliness and accuracy. YOLOv7 (18) proposed E-ELAN hierarchical feature fusion and auxiliary head training strategies. YOLOv8 (19), a representative work of the Ultralytics framework, adopted a decoupled detection head and anchor-free design, further improving feature extraction efficiency. YOLOv9 (20) addressed information loss in deep supervision via Programmable Gradient Information (PGI) and the Generalized Efficient Layer Aggregation Network (GELAN). YOLOv10 (21) introduced NMS-free training and a consistent dual-assignment strategy, enabling real-time end-to-end detection. YOLOv11 (22) and YOLOv12 (23) have iteratively advanced attention mechanisms and convolutional designs, respectively.

Although YOLO-series methods have seen rapid development in general scenarios, their application to lower limb fracture X-ray images still faces challenges such as insufficient extraction of multi-scale fracture features and low contrast at bone-soft tissue boundaries. Therefore, this paper aims to develop a fracture-specific detection method for lower limb fractures based on the YOLO approach.

### 2.2 Fracture detection

Fracture detection is an important direction in medical image analysis (24). Early CAD systems mostly relied on handcrafted features combined with traditional machine learning classifiers. These methods were limited by their feature representation capacity and exhibited poor robustness to the variability in fracture morphology (25).

With the advancement of deep learning, convolutional neural networks have gradually become the mainstream approach for X-ray fracture detection. For clinical emergency scenarios such as fracture detection, the balance between real-time performance and accuracy is particularly critical; thus, current research often focuses on the YOLO series. For example, Wang et al. (25) explored the performance of YOLO-series algorithms for fracture detection, highlighting the advantages of deep learning architectures. Qiu et al. (26) proposed X-YOLO, a novel wrist fracture detection method based on dynamic feature enhancement and lightweight design. Du et al. (9) introduced ASC-YOLO, a novel fracture detection method that effectively addresses the issue of missed small-target fractures. However, existing methods have been less studied for lower limb fractures, and research specifically targeting the multi-scale variation and ambiguous foreground-background boundaries of lower limb fractures is even scarcer. Therefore, this study develops MFRNet to address the challenges in lower limb fracture detection, further improving detection accuracy.

## 3 Methods

### 3.1 Overview

**Figure 1:**
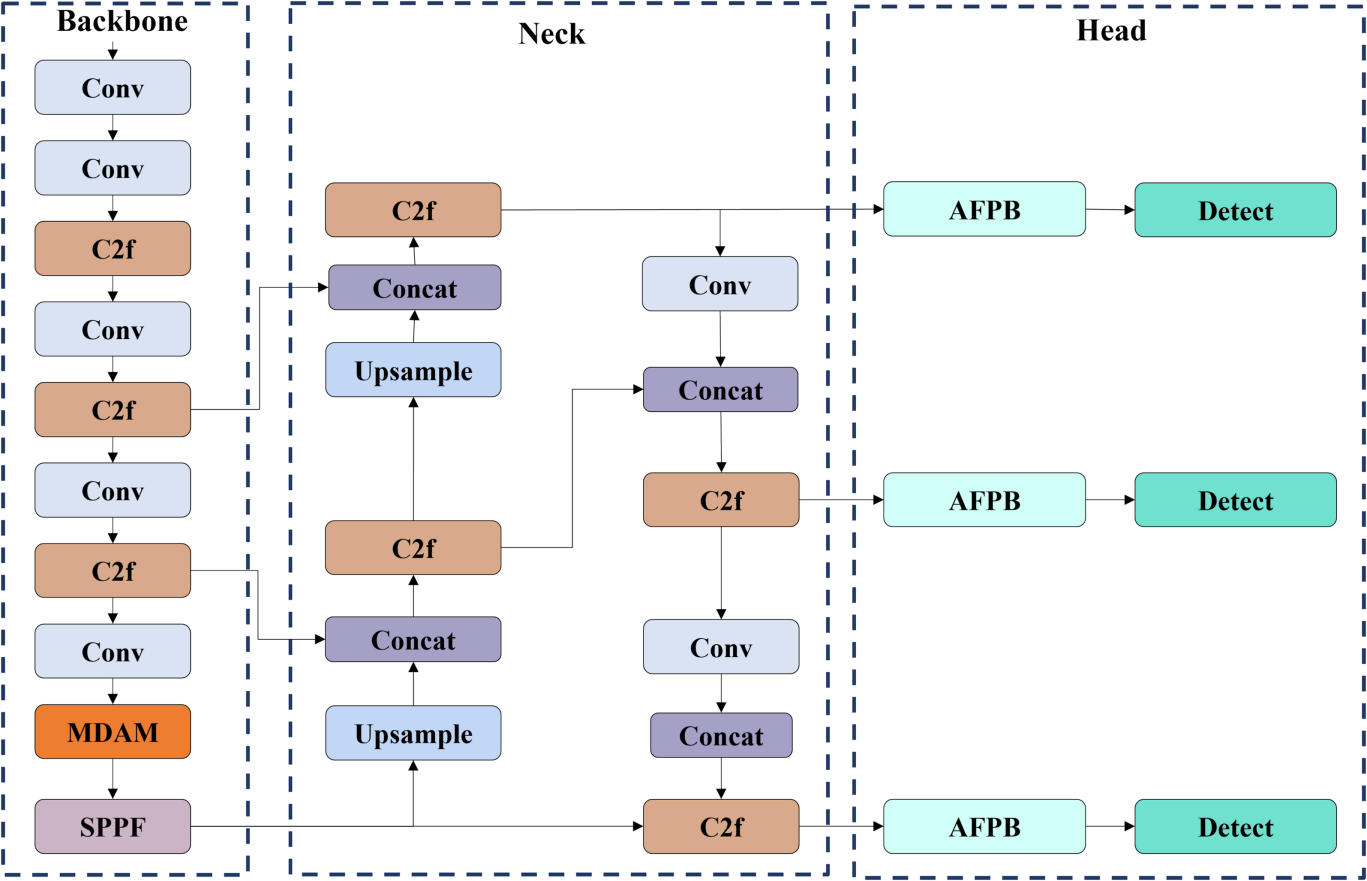
Architecture illustration of our MFRNet.

To address the challenges of large variations in fracture lesion scales and ambiguous boundaries between fracture foreground and background in lower limb X-ray images, this paper proposes a multi-scale feature refinement network, termed MFRNet (Multi-scale Feature Refinement Network). The overall architecture is illustrated in Figure 1. MFRNet is built upon the YOLOv8 framework and consists of three components: Backbone, Neck, and Head. To enhance the representation capability of fracture features, we design two novel modules: the Adaptive Feature Perception Block (AFPB) and the Multi-Scale Dilated Attention Module (MSDAM).

Specifically, for the input X-ray image $\;I\in {\mathbb{R}}^{3\times H\times W},$ the backbone extracts multi-scale features through progressive downsampling. In the last C2f block of the backbone, we replace the original Bottleneck with the MSDAM module to enlarge the effective receptive field and capture multi-scale contextual information. This replacement is applied to the high-level semantic features of the P5 layer.


$$F_{3},F_{4},F_{5}=\text{Backbone}(I)$$ (1)

where $F_{3},F_{4},F_{5}$ correspond to the feature maps of the P3, P4, and P5 layers, respectively. The neck network adopts a Path Aggregation Network (PANet) architecture, which employs bidirectional feature fusion (both top-down and bottom-up) to enhance information flow across different levels.


$${\hat{F}}_{3},{\hat{F}}_{4},{\hat{F}}_{5}=\text{Neeck}(F_{3},F_{4},F_{5})$$ (2)

To improve the sensitivity of each detection head to fracture regions, we insert an AFPB module in front of each of the three detection heads to perform adaptive feature extraction on the fused features.


$$\begin{array}{c} F_{i}^{\text{refined}}=F_{\text{AFPB}}({\hat{F}}_{i}),i\in \{3,4,5\} \end{array}$$ (3)

Finally, the detection heads output the bounding box and class of the fracture target based on multi-scale features:


$$\begin{array}{c} {\left\{b_{j},c_{j}\right\}}_{j=1}^{N}=\text{Detect}(F_{3}^{\text{refined}},F_{4}^{\text{refined}},F_{5}^{\text{refined}}) \end{array}$$ (4)

where $b_{j}\in {\mathbb{R}}^{4}$ represents the coordinates of the prediction box, $c_{j}$ is the corresponding confidence score and category, and *N* is the number of fractures detected.

### 3.2 Adaptive feature perception block

**Figure 2:**
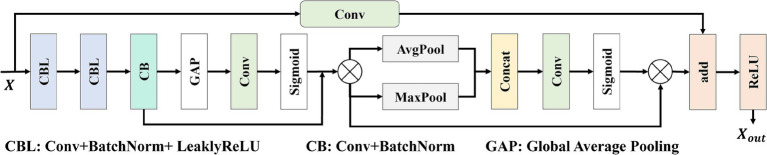
Architecture illustration of AFPB.

To address the challenge posed by the blurred boundary between fractures and their background, we have designed the AFPB, whose architecture is shown in Figure 2. By employing multi-level feature refinement and adaptive recalibration, the AFPB highlights fracture features while suppressing background noise, thereby enhancing the model’s ability to extract and distinguish features effectively.

Specifically, for the given input feature $X\in {\mathbb{R}}^{C_{\text{in}}\times H\times W}$ , we first use a 1 × 1 convolution to reduce the number of channels to an intermediate dimension $C_{\mathrm{mid}}$ , thereby lowering computational complexity ( $X^{\prime }$ ). Next, we employ a 3 × 3 convolution to extract spatial features( $X^{\prime \prime }$ ), and finally, another 1 × 1 convolution is used to expand the number of channels back to the output dimension $C_{\text{out}}$ . To enhance the model’s nonlinear expressiveness, each convolution is followed by batch normalization and a LeakyReLU activation function. The above transformation can be formally expressed as Equations (5–7):


$$\begin{array}{c} X^{\prime }=\sigma (\mathrm{BN}(\mathrm{Con}v_{1\times 1}^{C_{\mathrm{mid}}}(X))) \end{array}$$ (5)

$$\begin{array}{c} X^{\prime \prime }=\sigma (\mathrm{BN}(\mathrm{Con}v_{3\times 3}^{C_{\mathrm{mid}}}(X^{\prime }))) \end{array}$$ (6)

$$\begin{array}{c} X_{\text{bottle}}=\mathrm{BN}(\mathrm{Con}v_{1\times 1}^{C_{\text{out}}}(X^{\prime \prime })) \end{array}$$ (7)

where *σ*(·) is LeakyReLU activation function, BN(·) is batch normalization.

Building on the $X_{\text{bottle}}$ , we further apply serial channel attention and spatial attention to enhance the model’s focus on fracture regions. Channel attention adaptively recalibrates the importance of each feature channel; its computation process is shown in Equation (8):


$$\begin{array}{c} A_{c}(X)=\sigma_{c}(\mathrm{MLP}(\mathrm{GAP}(X_{\text{bottle}}))) \end{array}$$ (8)

where GAP represents global average pooling, MLP refers to a multilayer perceptron with a single hidden layer, and $\sigma_{c}$ represents the Sigmoid activation function. The feature after channel attention weighting is given by Equation (9):


$$\begin{array}{c} X^{\prime }=A_{c}(X_{\text{bottle}})⊗X_{\text{bottle}} \end{array}$$ (9)

where ⊗ represents element-wise multiplication.

Spatial attention compresses the features along the channel dimension to highlight the spatial location of the fracture. Specifically, $X^{\prime }$ is subjected to average pooling and max pooling, respectively, yielding two 1 × H × W feature maps. These are concatenated along the channels, then passed through a 7 × 7 convolution to generate a single-channel spatial weight map, which is then passed through a Sigmoid activation function to obtain the final attention output $A_{s}$ , as shown in Equations 10 and 11.


$$\begin{array}{c} A_{s}=\sigma_{s}(\mathrm{Con}v_{7\times 7}(\text{Concat}(\text{AvgPool}(X^{\prime }),\text{MaxPool}(X^{\prime })))) \end{array}$$ (10)

$$\begin{array}{c} X_{\text{attn}}=A_{s}⊗X^{\prime } \end{array}$$ (11)

To ensure stable gradient propagation, AFPB introduces residual connections. When the input and output dimensions are consistent and the stride is 1, the residual branch acts as an identity mapping. When downsampling or a change in the number of channels is required, the residual branch adjusts the dimension via a 1 × 1 convolution, as shown in Equation (12):


$$\begin{array}{c} X_{\mathrm{res}}=\{\begin{array}{c} X,\text{if}\;s=1\;\text{and}\;C_{\text{in}}=C_{\text{out}}\;\\ \mathrm{Con}v_{1\times 1}^{C_{\text{out}}}(X),\;\;\text{otherwise} \end{array} \end{array}$$ (12)

Ultimately, the output of AFPB is given by Formula (13):


$$\begin{array}{c} X_{\text{out}}=\text{ReLU}\;(X_{\mathrm{at}tn}+X_{\mathrm{res}}) \end{array}$$ (13)

### 3.3 Multi-scale dilated attention module

**Figure 3:**
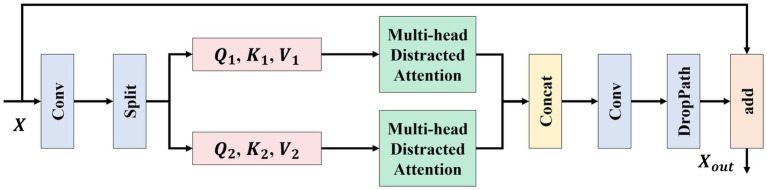
Architecture illustration of MSDAM.

To enhance the model’s ability to capture long-range dependencies and multi-scale contextual information, we introduce the Multi-Scale Dilated Attention Module (MSDAM) (30), whose architecture is shown in Figure 3. Unlike standard convolution operations with a fixed receptive field, MSDAM integrates dilated convolutions into the multi-head attention framework, enabling the model to perceive fractures at different scales without increasing the number of parameters.

Specifically, given an input feature map $X\in {\mathbb{R}}^{C\times H\times W}$ , MSDAM first applies a 1 × 1 convolution to generate the projections of the query, key, and value, as shown in Equation (14):


$$\begin{array}{c} Q,K,V=\mathrm{Con}v_{1\times 1}^{3C}(X) \end{array}$$ (14)

where


$$Q,K,V\in {\mathbb{R}}^{C\times H\times W}.$$

Next, the channel dimensions are partitioned into two groups corresponding to different expansion rates. Let $r=\{r_{1},r_{2}\}$ denote the set of expansion rates, where $r_{1}=1,r_{2}=2$ . The feature map partition is shown in Equation (15):


$$\begin{array}{c} Q=[Q_{1},Q_{2}],K=[K_{1},K_{2}],V=[V_{1},V_{2}] \end{array}$$ (15)

where each


$$Q_{i},K_{i},V_{i}\in {\mathbb{R}}^{(C/M)\times H\times W}.$$

For the $i_{\mathrm{th}}$ group with an expansion rate of *rᵢ*, the dilated attention is calculated as shown in Equation (16):


$$\begin{array}{c} \mathrm{Att}n_{i}(Q_{i},K_{i},V_{i})=\text{Softmax}(\frac{Q_{i}\cdot \text{Unfol}d_{r_{i}}(K_{i})}{\sqrt{d}})\cdot \text{Unfol}d_{r_{i}}(V_{i}) \end{array}$$ (16)

where $d=C/(2\cdot N_{\text{head}})$ represents the dimension of each head, $N_{\text{head}}$ is the number of attention heads per group, $\text{Unfol}d_{r}(\cdot )$ denotes a sliding window unfolding operation with a kernel size of *k* = 3 and a dilation rate of *r*.

To stabilize training and enhance representational capacity, multi-head attention is employed within each dilation group, as shown in Equations (17) and (18):


$$\begin{array}{c} \text{MHAtt}n_{i}(Q_{i},K_{i},V_{i})=\text{Concat}({\text{head}}_{1},\ldots .,{\text{head}}_{N_{\text{head}}})W_{i}^{O} \end{array}$$ (17)

$$\begin{array}{c} {\text{head}}_{j}=\mathrm{Att}n_{i}(Q_{i}^{\left(j\right)},K_{i}^{\left(j\right)},V_{i}^{\left(j\right)}) \end{array}$$ (18)

The outputs of both groups are concatenated along the channel dimension and projected, as shown in Equation (19):


$$\begin{array}{c} \hat{X}=\mathrm{Con}v_{1\times 1}(\text{Concat}(\text{MHAtt}n_{1},\text{MHAtt}n_{2})) \end{array}$$ (19)

Finally, residual connections and a random drop path (DropPath) are employed to facilitate gradient flow and prevent overfitting. The final output is given by Equation (20):


$$\begin{array}{c} X_{\text{out}}=X+\text{DropPath}(\hat{X}) \end{array}\;$$ (20)

## 4 Results and discussion

### 4.1 Experiments setup

To ensure the fairness of all comparative experiments and the reproducibility of the results, this section details the hardware platform, software environment, and specific training parameters used for all models.

### 4.2 Hardware and frameworks

All experiments were conducted on the same computing platform: the operating system was Ubuntu 20.04, the CPU was an AMD EPYC 9554, the GPU was an NVIDIA GeForce RTX 4090 (with 24 GB of video memory), and the system memory was 128 GB. The deep learning framework used was PyTorch 1.12.0, with CUDA version 11.6 and cuDNN version 8.3. All YOLO-series models were trained using the default configurations from their respective official code repositories, under identical experimental conditions.

### 4.3 Training parameters

For a fair comparison, all models were configured with uniform hyperparameters. The specific parameter settings are shown in Table 1.

**Table 1:** Experimental parameter settings.

Parameter	Setting
Image size	640 × 640
Initial learning rate	0.01
momentum	0.937
decay	0.0005
Batch size	16
optimizer	SGD
Workers	8
IoU	0.7
Epoch	100

### 4.4 Dataset

The lower-limb fracture X-ray image dataset used in this study was collected from the Department of Orthopedics at Handan No.1 Hospital, with a time span from April 2021 to March 2026. The original dataset comprises a total of 1,924 X-ray images, covering areas such as the femur and tibia-fibula. All images were independently annotated by two attending orthopedic physicians, with the annotation category being “fracture.”

To ensure data diversity and the model’s generalization capability, we performed data augmentation on the original training set using a random mixing augmentation strategy that includes: (1) brightness and contrast adjustments, with brightness adjusted within the range [−30, +30] and contrast adjusted within the range [0.7, 1.3]; (2) random rotation, with rotation angles ranging from [−45°, +45°]; (3) random cropping, which generates multiple random square masking regions; and (4) horizontal flipping.

To prevent data leakage, we chose to perform data splitting before data augmentation. After comprehensively evaluating existing data splitting methods, this study conducted experiments using two data splitting settings to fully validate the model’s effectiveness.

In Setting 1, the original data was first split into a training set and a validation set in a 7:3 ratio, and then data augmentation was applied to each set separately. The augmented data is twice the size of the original data, with 2,692 images in the training set and 1,156 images in the validation set.

In Setting 2, the original data is first divided into training, validation, and test sets in a 70:15:15 ratio, and data augmentation is performed only on the training set. The validation and test sets contain only the original images, while the augmented training set contains twice as many images as the original training set.

### 4.5 Evaluation metric

To comprehensively evaluate the detection performance of each model, we adopted commonly used evaluation metrics in the object detection field, including precision (P), recall (R), mean Average Precision (mAP), and the number of parameters (#Params). The definitions of these metrics are as follows.

Precision represents the proportion of samples predicted as positive cases that are actually positive, as shown in Equation (21):


$$\begin{array}{c} \text{Precision}=\frac{\mathrm{TP}}{\mathrm{TP}+\mathrm{FP}} \end{array}$$ (21)

Recall represents the proportion of all true positive cases that are correctly predicted, as shown in Equation (22):


$$\begin{array}{c} \text{Recall}=\frac{\mathrm{TP}}{\mathrm{TP}+\mathrm{FN}} \end{array}$$ (22)

Among them, TP, FP, and FN represent the numbers of true positives, false positives, and false negatives, respectively. The average precision (AP) is calculated by determining the area under the Precision-Recall curve, while the mean Average Precision (mAP) is the average of AP values across all categories. This paper reports two metrics: mAP@0.5 (with an IoU threshold of 0.5) and mAP@0.5:0.95 (with an IoU threshold ranging from 0.5 to 0.95 in steps of 0.05). The number of parameters, measured in millions (M), refers to the total number of trainable parameters in the model and is used to assess the model’s lightweight nature.

### 4.6 Comparison experiments

To validate the effectiveness of the MFRNet proposed in this paper, we conducted a comprehensive comparison between our method and current state-of-the-art object detection algorithms under the same dataset and training settings. We compared our MFRNet with nano, small, and medium variants of mainstream YOLO versions because these sizes are most relevant to our lightweight model (3.8 M parameters). Nano variants represent the most compact models suitable for edge deployment, while small and medium variants show that larger models do not necessarily yield better performance on this task. Large and extra-large variants were omitted due to their impractical parameter counts. The results of the comparative experiments are shown in Table 2 (Setting 1) and Table 3 (Setting 2).

**Table 2:** Comparison with the advanced methods in the setting 1.

Model		Precision	Recall	mAP	mAP50-95	#P
YOLO11 (22)	YOLO11n	85.7	82.2	87.3	50.5	2.6 M
	YOLO11s	86.2	83.6	88.3	51.9	9.4 M
	YOLO11m	85.0	81.1	86.6	51.3	20.0 M
YOLOv10 (21)	YOLOv10n	83.6	78.3	85.5	49.3	2.7 M
	YOLOv10s	84.1	80.5	86.1	50.4	8.0 M
	YOLOv10m	88.4	78.2	85.0	49.5	16.5 M
YOLOv9 (20)	YOLOv9t	86.2	79.7	86.4	50.4	2.0 M
	YOLOv9s	83.8	82.6	87.7	51.9	7.2 M
	YOLOv9m	84.6	84.3	87.7	51.2	20.0 M
YOLOv8 (19)	YOLOv8n	86.4	80.8	87.2	49.5	3.0 M
	YOLOv8s	85.7	84.2	87.3	50.1	11.1 M
	YOLOv8m	86.2	80.1	87.5	51.6	25.8 M
YOLOv6 (17)	YOLOv6n	85.2	77.2	84.9	46.9	4.2 M
	YOLOv6s	85.3	81.1	86.2	46.9	16.3 M
	YOLOv6m	83.5	80.2	85.2	46.7	52.0 M
YOLOv5 (16)	YOLOv5n	84.5	81.6	85.6	48.9	2.5 M
	YOLOv5s	84.7	81.6	86.4	50.1	9.1 M
	YOLOv5m	86.5	80.9	86.3	50.5	25.0 M
YOLO26 (27)	YOLO26n	83.7	79.3	84.9	48.3	2.4 M
	YOLO26s	86.6	80.3	85.3	48.1	9.9 M
	YOLO26m	84.3	80.3	85.1	48.9	21.8 M
YOLO12 (23)	YOLOv12n	83.9	79.3	84.8	47.7	2.6 M
	YOLOv12s	84.6	81.2	84.9	47.6	9.3 M
	YOLOv12m	86.6	79.4	85.7	48.5	20.2 M
Faster-RCNN (11)		83.1	80.9	85.2	46.9	41.2 M
YOLOv8-AM (28)		86.4	83.2	87.2	51.5	3.0 M
RT-DETR-L (29)		89.3	83.4	86.0	44.7	32.8 M
MFRNet		87.5	84.2	89.1	52.2	3.8 M

**Table 3:** Results of stability experiments in the setting 2.

Data	Metric	Baseline	YOLO11n (22)	Faster-RCNN (11)	YOLO26n (27)	RT-DETR (29)	MFRNet
Val	Precision	83.8	83.6	83.9	87.4	86.8	87.3
	Recall	82.7	85.1	78.1	77.8	82.7	83.7
	mAP	85.8	87.1	84.3	85.2	86.4	89.0
	mAP50-95	50.8	50.2	49.0	49.1	50.2	52.6
Test	Precision	84.4	86.6	81.8	88.2	88.0	87.7
	Recall	84.5	85.3	83.7	77.7	81.2	83.3
	mAP	88.0	89.4	86.8	87.1	88.7	90.2
	mAP50-95	53.5	53.9	51.0	51.4	53.0	55.2

**Figure 4:**
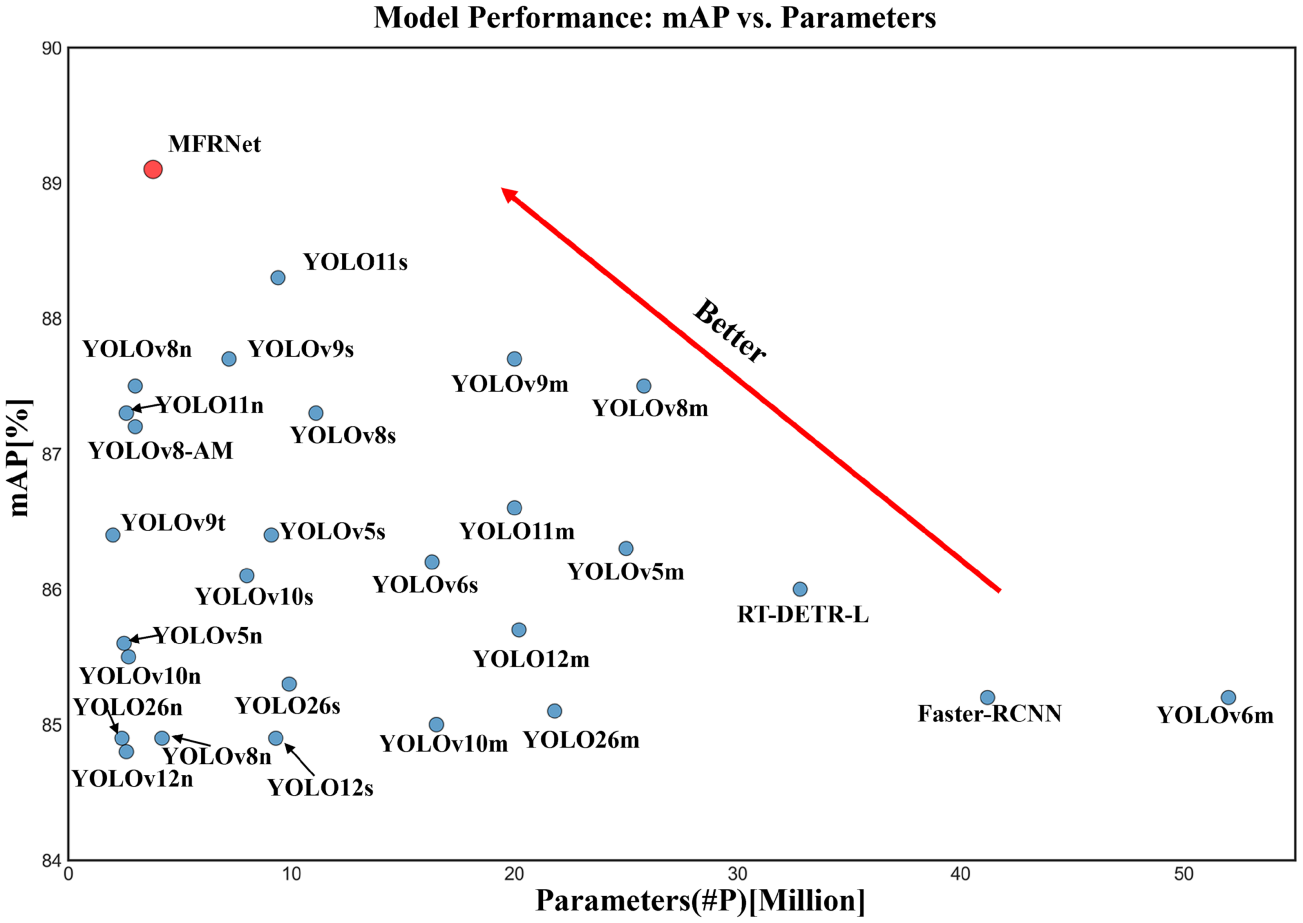
Visualization of model comprehensive performance comparison.

As shown in Table 2, the MFRNet proposed in this paper achieves the highest mAP (89.1%) and mAP50-95 (52.2%) with only 3.8 M parameters. Moreover, its precision and recall also outperform most of the compared models. Compared to the baseline YOLOv8n, MFRNet exhibits a 1.1% improvement in precision, a 1.9% increase in mAP, a 2.7% enhancement in mAP50-95, and a 2.4% boost in recall—all while increasing the parameters by only 0.8 M. These results demonstrate that the AFPB and MSDAM modules effectively enhance the model’s ability to detect fracture targets. It is worth noting that some models with larger parameter sizes (such as Faster-RCNN, RT-DETR, YOLOv8m and YOLOv9m) did not achieve better performance than MFRNet, indicating that simply increasing the depth and width of the network is not the optimal choice for the specific task of lower-limb fracture detection. By introducing attention-guided feature refinement and multi-scale dilated attention at key locations, MFRNet strikes an excellent balance between parameter efficiency and detection accuracy. Furthermore, MFRNet also performs best on the more stringent mAP50-95 metric, indicating that its improvement in localization accuracy is particularly significant, which holds clinical value for precisely measuring fracture displacement distances. Figure 4 shows a performance comparison between different object detection models in terms of both parameters and mAP. Qualitative results indicate that MFRNet achieves the highest mAP even with an extremely small number of parameters, significantly outperforming other models. In contrast, models such as YOLOv8m and YOLOv9m, despite having a larger number of parameters, deliver lower mAP performance compared to MFRNet.

In Setting 2, we compared models’ performance on both the validation set and the test set. Quantitative results show that MFRNet achieved the highest mAP and mAP50-95 on both the validation and test sets. Compared to baseline models, MFRNet demonstrated comprehensive performance improvements on the validation set, with a 3.5% increase in Precision, a 1.0% increase in Recall, a 3.2% increase in mAP, and a 1.8% increase in mAP50-95. On the test set, MFRNet also achieved significant improvements over the baseline, with a 3.3% increase in Precision, a 2.2% increase in mAP, and a 1.7% increase in mAP50-95. The comprehensive experimental results under Setting 2 further validate the effectiveness of the method proposed in this study.

To assess the stability of our method, all experiments were repeated three times. The results reported in Table 4 are presented as mean ± standard deviation (SD) across the three runs. As shown in Table 2, MFRNet achieves 87.77 ± 0.64% precision, 84.17 ± 0.75% recall, 89.20 ± 0.36% mAP, and 52.03 ± 0.21% mAP50-95. The small SD values indicate that MFRNet yields consistent and stable detection performance.

**Table 4:** Results of stability experiments.

Model	Precision	Recall	mAP	mAP50-95	#P
MFRNet-1	87.5	84.2	89.1	51.8	3.8 M
MFRNet-2	87.3	84.9	89.6	52.1	3.8 M
MFRNet-3	88.5	83.4	88.9	52.2	3.8 M
MFRNet	87.77 ± 0.64	84.17 ± 0.75	89.20 ± 0.36	52.03 ± 0.21	3.8 M

**Figure 5:**
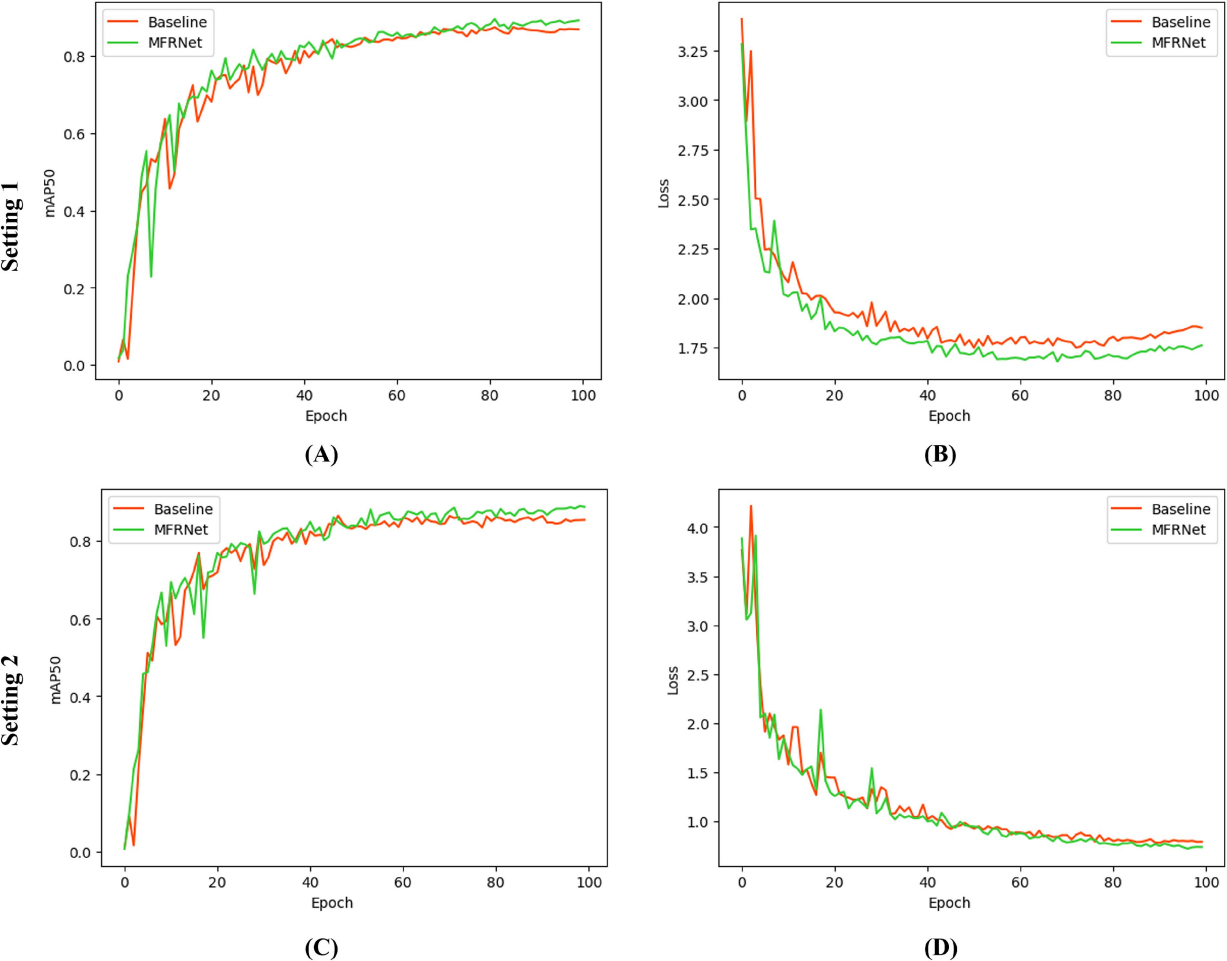
Comparison of Training Processes. **(A)** shows the mAP comparison in setting 1, and **(B)** shows the loss comparison in setting 1, **(C)** shows the mAP comparison in setting 2, and **(D)** shows the loss comparison in setting 2.

Figure 5 shows a comparison of the mAP and loss curves between MFRNet and the baseline YOLOv8n during the training process. As shown in Figure 5(A) and Figure 5(C), MFRNet exhibits a faster growth rate in mAP during the early stages of training, achieves a higher final convergence value, and has smaller oscillations. This indicates that the two modules help accelerate convergence and stabilize training. The loss curve in Figure 5(B) and Figure 5(D) reveals that MFRNet’s total loss decreases more rapidly and ultimately reaches a lower final value than the baseline, demonstrating the optimization effect of residual connections and the attention mechanism on gradient flow.

### 4.7 Ablation study

To quantitatively evaluate the contributions of the AFPB and MSDAM modules separately, we designed an ablation study. Using YOLOv8n as the baseline model, we gradually added the AFPB and MSDAM modules. All ablation models used the same training configuration, and the results are shown in Table 5.

**Table 5:** Results of ablation experiments on the validation set.

Model	AFPB	MSDAM	Precision	Recall	mAP
Baseline	×	×	86.4	80.8	87.2
+AFPB	√	×	86.9	83.0	88.7
+MSDAM	×	√	88.4	82.5	88.2
MFRNet	√	√	87.5	84.2	89.1

As shown in Table 4, after adding the AFPB module alone, the model’s mAP increased from 87.2 to 88.7%, and the recall rate significantly improved from 80.8 to 83.0%. This indicates that the AFPB module, with its bottleneck structure and lightweight dual-attention mechanism, effectively suppresses background noise in X-ray images, enabling the model to capture more genuine fracture regions and thereby enhancing its detection accuracy.

After adding the MSDAM module separately, the mAP improved to 88.2%, and the precision increased significantly from 86.4 to 88.4%. This indicates that MSDAM, through multi-scale dilated attention, has expanded the receptive field, enabling the model to better distinguish between bone edges and soft-tissue shadows, thereby reducing false positives and misclassifications.

When AFPB and MSDAM were used together, MFRNet achieved the best mAP (89.1%) and recall (84.2%). The comprehensive experimental results indicate that AFPB and MSDAM demonstrate significant synergistic benefits, effectively improving model performance by enhancing feature responses in fracture regions and capturing multiscale contextual information.

### 4.8 Visual comparison

**Figure 6:**
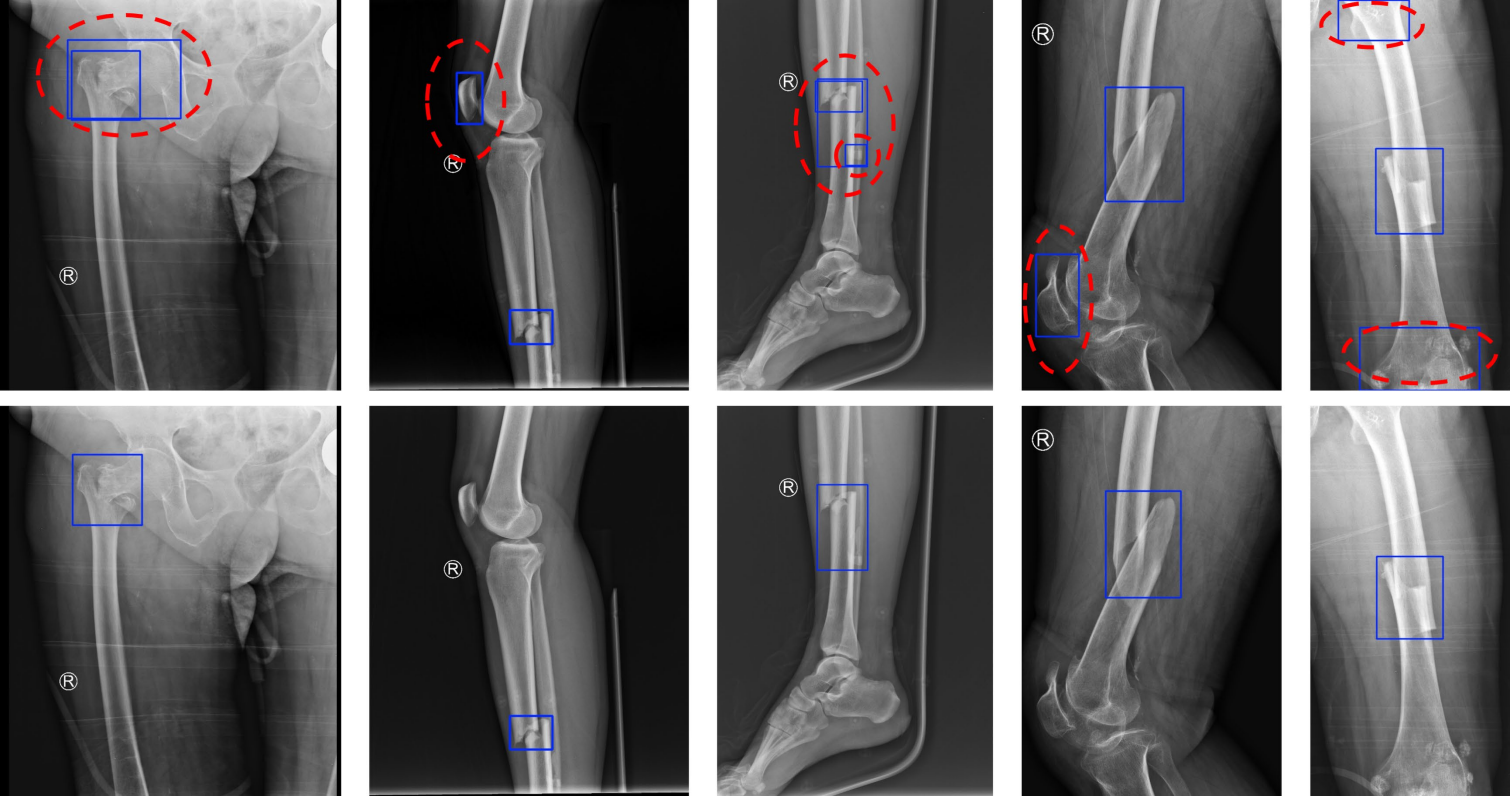
Visual comparison of detection results. The top row is baseline, the bottom row is MFRNet.

To intuitively demonstrate the detection performance of MFRNet, we selected representative samples for visual comparison. The visualization results are shown in Figure 6. As can be clearly observed from the comparison, the Baseline model exhibited noticeable misdetections and inaccurate regression in several samples, failing to precisely identify the fracture regions. In contrast, MFRNet was able to accurately localize and detect the target regions, demonstrating a clear advantage. Qualitative results indicate that MFRNet significantly outperforms the Baseline model in terms of robustness and accuracy under complex scenarios, further validating its superior detection performance.

### 4.9 Limitations and future directions

Although the MFRNet proposed in this paper achieves significant performance improvements in the task of lower-limb fracture detection, it still has certain limitations. First, the current model has been trained and validated exclusively on lower-limb X-ray images and has not been tested on other anatomical regions (such as upper limbs and spine) or imaging modalities (such as CT and MRI), so its generalization ability remains to be further verified. Second, the MFRNet has 3.8 million parameters, which is an increase over YOLOv8n’s 3.0 million. Although this increase is relatively small, in extremely lightweight mobile deployment scenarios, further pruning or quantization may be necessary.

In the future, we will embark on explorations in the following areas: (1) validating our model on larger-scale, multi-center X-ray datasets and exploring cross-modal transfer learning to enhance the model’s generalizability; (2) integrating fracture classification information (such as the AO classification system) to design a multi-task learning framework that simultaneously performs fracture detection and classification; (3) introducing semi-supervised or unsupervised learning methods to reduce reliance on finely annotated data; (4) deploying MFRNet into real-world clinical decision-support systems for prospective clinical validation, and evaluating its effectiveness in improving physicians’ diagnostic efficiency.

## 5 Conclusion

In this paper, we propose a multi-scale feature refinement network, MFRNet, to address the challenges of large variations in lesion scales and ambiguous boundaries between fracture foreground and background in lower limb X-ray images. MFRNet is built upon the YOLOv8 framework and achieves efficient feature extraction and precise localization through two novel modules. Specifically, AFPB effectively enhances fracture-related features while suppressing background noise, thereby improving feature discriminability of fracture regions. MSDAM expands the receptive field via parallel dilated convolutions with different dilation rates and a multi-head attention mechanism, thus enhancing the model’s multi-scale context extraction capability. Comprehensive experimental results demonstrate that MFRNet achieves 89.1% mAP and 52.2% mAP50-95 with only 3.8 M parameters, significantly outperforming all compared methods and exhibiting favorable detection performance and clinical practical value. This study provides a lightweight yet efficient solution for computer-aided diagnosis of lower limb fractures. Future work will continue to explore multi-center generalization validation, multitask learning for fracture classification, and mobile deployment.

## Data Availability

The raw data supporting the conclusions of this article will be made available by the authors, without undue reservation.
